# Cell adhesion to collagen promotes leukemia resistance to doxorubicin by reducing DNA damage through the inhibition of Rac1 activation

**DOI:** 10.1038/s41598-019-55934-w

**Published:** 2019-12-19

**Authors:** Dalila Naci, Sofiane Berrazouane, Frédéric Barabé, Fawzi Aoudjit

**Affiliations:** 1 Centre de recherche du CHU de Québec-Université Laval, Axe des maladies infectieuses et immunitaires, Québec, Canada; 2 Département de Médicine, Faculté de Médecine, Université Laval, Québec, Canada; 3 Département de Microbiologie-infectiologie et Immunologie, Faculté de Médecine, Université Laval, Québec, Canada; 4 Present Address: The Hospital for Sick Children, University of Toronto, Toronto, Canada

**Keywords:** Cancer microenvironment, Extracellular matrix, Integrins, Apoptosis, Acute lymphocytic leukaemia

## Abstract

Chemoresistance is a major hurdle in anti-cancer therapy. Growing evidence indicates that integrin-mediated cell adhesion to extracellular matrix plays a major role in chemoresistance. However, the underlying mechanisms are not fully understood. We have previously shown that the collagen-binding integrin α2β1 promoted doxorubicin resistance in acute T cell lymphoblastic leukemia (T-ALL). In this study, we found that acute myeloid leukemia (AML) cell lines also express α2β1 integrin and collagen promoted their chemoresistance as well. Furthermore, we found that high levels of α2 integrin correlate with worse overall survival in AML. Our results showed that doxorubicin-induced apoptosis in leukemic cells is associated with activation of Ras-related C3 botulinum toxin substrate 1 (Rac1) and that collagen inhibited this pathway. The protective effect of collagen is associated with the inhibition of Rac1-induced DNA damage as evaluated by the comet assay and the phosphorylated levels of histone H2AX (γ-H2AX). Together these results show that by inhibiting pro-apoptotic Rac1, α2β1 integrin can be a major pathway protecting leukemic cells from genotoxic agents and may thus represent an important therapeutic target in anti-cancer treatment.

## Introduction

Integrins are α/β heterodimers that mediate cell-cell and cell-extracellular matrix (ECM) interactions. Integrin-mediated attachment to ECM is critical for cell invasion, cancer growth and metastasis^1^. In addition, integrins also regulate cell survival. Normal epithelial and endothelial cells undergo a form of cell death known as anoikis when cultured in suspension or on an inappropriate matrix protein^2^. Growing evidence indicates that integrin-ECM interactions are also involved in the survival and resistance of cancer cells to chemotherapy (chemo or drug resistance), which remains still a significant hurdle in anti-cancer therapies^3^. A major action of chemotherapy on cancer cells occurs via induction of apoptosis and thus understanding how integrins modulate chemotherapy-induced apoptosis will likely lead to more efficient therapies.

Cell adhesion can promote drug resistance via multiple mechanisms; by inhibiting the apoptotic signaling cascade, enhancing drug efflux and activating DNA repair. These mechanisms are often simultaneously deregulated in drug resistant cells. Integrin-ECM interactions inhibit chemotherapy-induced apoptosis of various cancer cell lines by directly upregulating B-cell lymphoma 2 (Bcl-2) pro-survival proteins and inhibiting pro-apoptotic Bcl-2 proteins^3–5^. In T-cell acute lymphoblastic leukemia (T-ALL), the collagen-binding integrin α2β1 promotes doxorubicin resistance by maintaining the levels of anti-apoptotic protein, myeloid cell leukemia-1 (Mcl-1) through the inhibition of the c-Jun N-terminal Kinase (JNK) activation^6^. β1 integrins have also been shown to enhance drug efflux in leukemic cells via the upregulation of drug transporters of the ATP Binding Cassette (ABC) superfamily^7,8^. Finally, it has been reported that integrins also promote DNA repair in cancer cells in response to DNA damaging drugs and to irradiation treatment^9–11^. Despite these findings, the mechanisms by which integrins promote cancer chemoresistance are not fully elucidated.

Ras-related C3 botulinum toxin substrate 1 (Rac1) belongs to the Rho family of small GTPases and plays a central role in cytoskeleton organization and migration and as such has been associated with cancer invasion^12,13^. Recent studies have attributed a role for Rac1 in apoptosis. Rac1 contributes to podocyte injury in chronic kidney disease^14^, β-amyloid peptide-induced neuronal death^15^ and to neuronal death during the ischemic stroke^16^. With regard to drug-induced apoptosis, the implication of Rac1 has mostly been studied in the cytotoxicity of doxorubicin in cardiomyocytes^17,18^. In these cells, Rac1 has been associated with DNA damage response to topoisomerase II inhibitors such as doxorubicin^19–21^. Rac1 enhances the interactions of topoisomerases II with their respective drugs, which then leads to the formation of double strands breaks and to the DNA damage response and induction of cell death^19–22^. A recent study reported that in cooperation with FLT3/ITD, Rac1 modulates the sensitivity of leukemic cells to chemotherapy via the regulation of DNA repair^23^. Despite these findings, the role of Rac1 in leukemic cell response to genotoxic drugs remains unclear.

Anthracyclines, among which is doxorubicin, are powerful anti-cancer agents and are part of the standard regimen in the treatment of acute leukemia^24^. Understanding how integrins regulate anthracycline-induced stress response and apoptosis has potential high value for cancer treatment in the clinic. In this study, we showed that the collagen/α2β1 integrin interaction promotes doxorubicin resistance in lymphoblastic and myeloid leukemic cells by reducing DNA damage through Rac1 inhibition. Thus targeting α2β1 integrin and/or the development of Rac1-independent genotoxic agents may enhance anti-cancer treatment.

## Results

### α2β1 integrin protects AML cells from doxorubicin-induced apoptosis and is associated with poor prognosis

**Figure 1:**
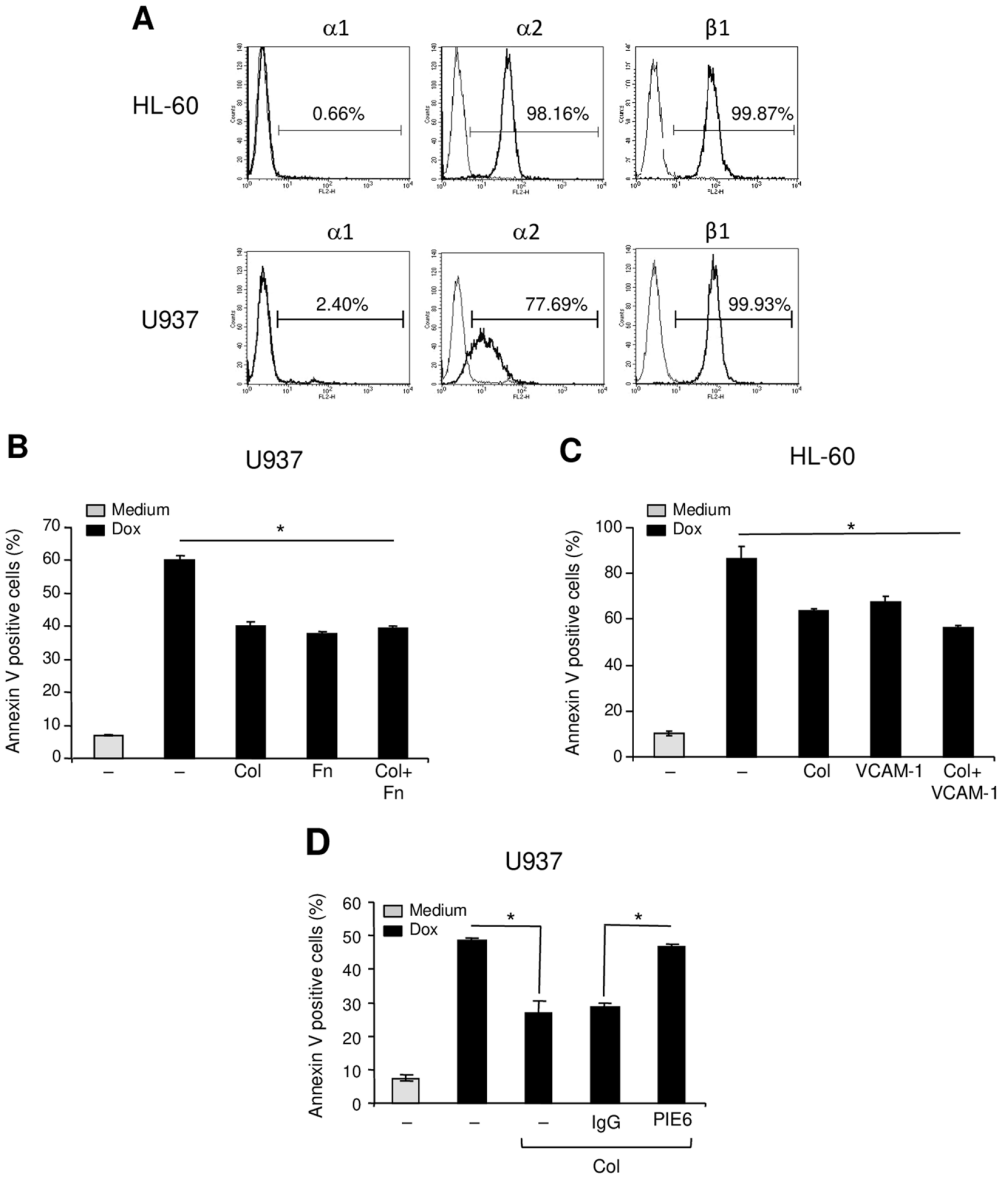
Collagen via α2β1 integrin protects AML cell lines from doxorubicin-induced apoptosis. (**A**) Flow cytometry analysis of α1, α2 and β1 integrin subunits expression on HL-60 and U937 cells. (**B**,**C**) Collagen reduces doxorubicin-induced apoptosis of U937 and HL-60 cells. The cells were cultured on BSA (–), collagen (Col), fibronectin (Fn) or on VCAM-1 as indicated for 2 h. Cells in suspension were washed and adherent cells were treated with 1 μM doxorubicin (Dox) for 24 h. Apoptosis was determined by annexin V staining and flow cytometry analysis. The results represent mean values ± SD from three independent experiments. *P < 0.05 between doxorubicin-treated samples cultured on collagen, fibronectin or VCAM-1 and doxorubicin-treated samples cultured on BSA (–). (**D**) α2 integrin blockade reverses the collagen protective effect. U937 cells were pretreated with 10 μg/ml of anti-α2 blocking antibody (PIE6) or with isotypic control IgG for 1 h before their culture on collagen. The cells were then treated with doxorubicin and apoptosis was determined by annexin V staining and flow cytometry analysis. The results represent mean values ± SD from three independent experiments. *P < 0.05.

We have previously shown that collagen-α2β1 integrin promotes doxorubicin resistance of human T-ALL cells^6^. To extend the role of α2β1 integrin in chemoresistance to additional types of leukemia, we examined its implication in acute myeloid leukemia (AML). We studied the role of collagen in the chemoresistance of well-characterized AML cell lines HL-60 and U937. We first examined the expression of the two major collagen-binding integrins α1β1 and α2β1 in AML cells. Both HL-60 and U937 express α2 integrin but not α1 integrin subunit and as expected, both cell lines express high levels of β1 integrin subunit (Fig. 1A). These data indicate that α2β1 but not α1β1 is the major collagen-binding integrin expressed on AML cells.

We then evaluated the role of α2β1 integrin in mediating HL-60 and U937 protection against doxorubicin-induced apoptosis. U937 and HL-60 cells cultured on collagen were significantly protected against doxorubicin-induced apoptosis. Apoptosis of U937 cells and HL-60 cells adhering to collagen were reduced by 33.34% and 25.3% respectively, comparing to cells cultured on BSA (Fig. 1B,C). Similar results were also obtained with the PLB-985 AML cell line (Supplementary Fig. S1). The collagen protective effect is similar to that obtained with cells adherent to vascular cell adhesion molecule 1 (VCAM-1) or to fibronectin, the ligands of α4β1 and α5β1 integrins previously implicated in chemoresistance of AML cells^25^. Simultaneous adhesion of AML cell lines to collagen + fibronectin or collagen + VCAM-1 did not enhance further their resistance to doxorubicin-induced apoptosis (Fig. 1B,C). The protective effect of collagen is mediated via α2 integrin as it is reversed by the use of a specific anti-α2 integrin blocking mAb (clone P1E6) (Fig. 1D). Together these results show that beyond T-ALL cells^6^, α2β1 integrin promotes doxorubicin resistance in AML cells suggesting that it can be a major pathway of leukemia chemoresistance.

**Figure 2:**
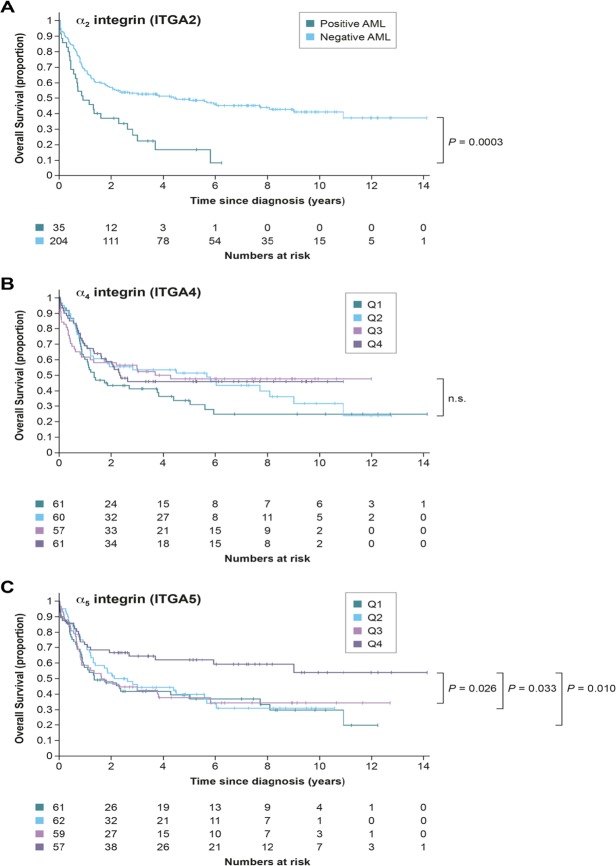
Overall survival of AML patients under 60 years old in the Leucegene cohort according to the mRNA expression of α2 integrin (**A**), α4 integrin (**B**) and α5 integrin (**C**). The mRNA expression levels for α4 and α5 integrins were divided into four quartiles (Q) with Q1 being the lowest expression level.

To determine whether these findings could have a clinical significance, we examined the correlation of α2 integrin levels with overall survival in the Leucegene AML cohort of patients (www.leucegene.ca). RNA sequencing data and clinical annotation for the cohort has already been reported^26^. Genes with TPM (Transcripts Per Kilobase Million) above 0.5 are generally detectable at the protein level and that cutoff was used to determine positive and negative α2 integrin AMLs. The 239 AML patients aged <60-year-old were included in the analyses. The results show that α2 integrin-positive AML patients (TPM ≥0.5) have a worse prognosis than those not expressing it (Fig. 2A, p = 0.0003).

Since α4β1 integrin, which binds to fibronectin and VCAM-1, and α5β1 integrin, which binds to fibronectin have been involved with the inhibition of chemotherapy-induced apoptosis in AML and in B cell malignancies^27–29^, we examined their association with clinical outcome in AML. AML samples expressed high mRNA levels of α4 integrin (TPM between 8.8 to 277.5, mean of 64.5) and α5 integrin (TPM between 0.20 to 237.7, mean of 47.1, with only 1 patient < 0.5). Since all AML were positive for both genes, we divided the samples into quartiles and looked at survival. α4 integrin expression levels have no impact on survival in our cohort while high expression of α5 integrin (4^th^ quartile) confers a statistically significant better prognosis when compared to all other quartiles (Fig. 2B,C).

### Rac1 is implicated in doxorubicin-induced apoptosis of leukemic cells

**Figure 3:**
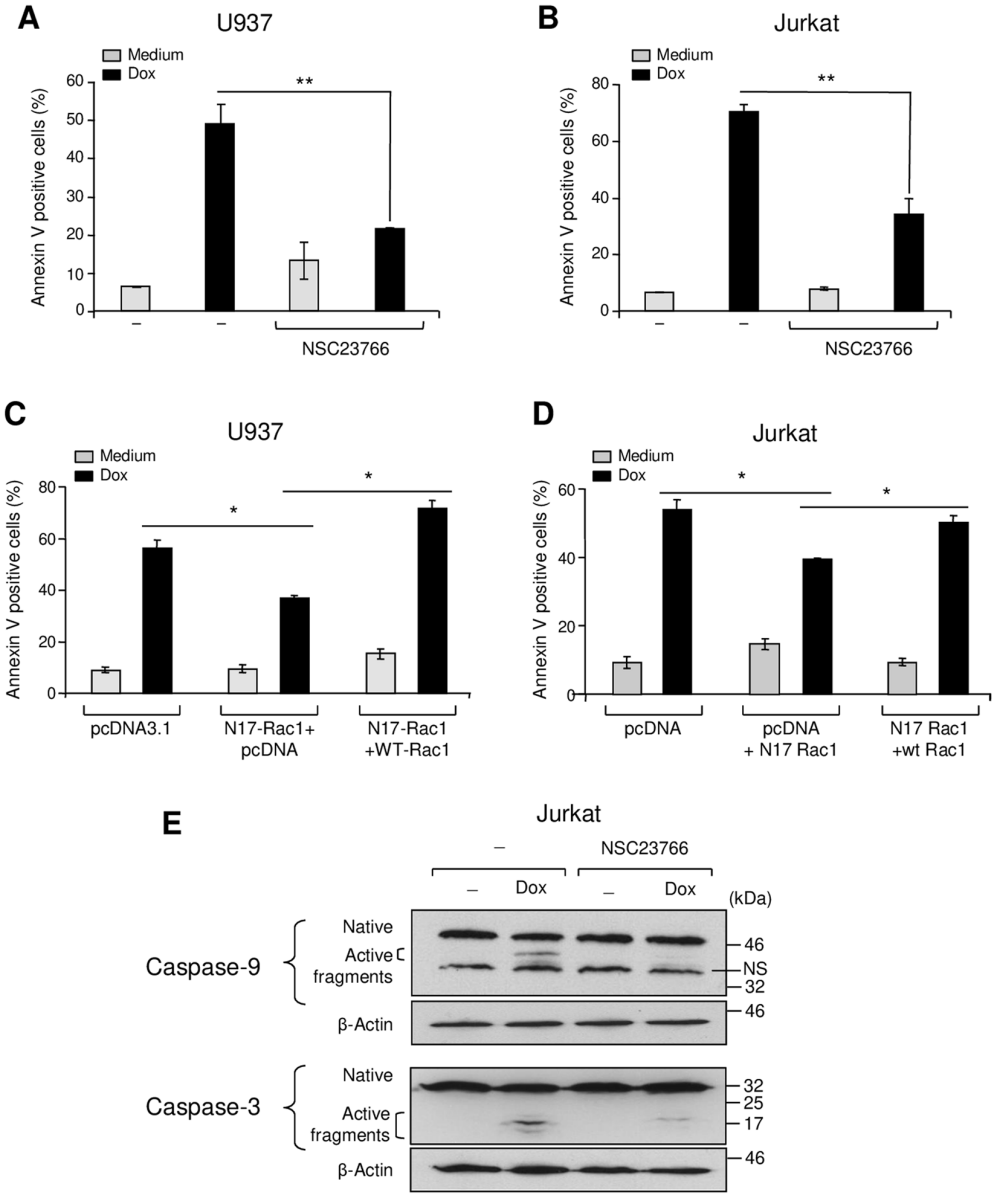
Doxorubicin-induced apoptosis in leukemic cells is dependent on Rac1. (**A**,**B**) The Rac1 inhibitor reduces doxorubicin-induced apoptosis in U937 and Jurkat cells. The cells were pretreated with the specific Rac1 inhibitor (NSC23766, 10 μM, 1 h), before their treatment with doxorubicin (Dox) for 24 h. Apoptosis was determined by annexin V staining and flow cytometry analysis. (**C**,**D**) Overexpression of the Rac1 dominant negative form N17Rac1 inhibits doxorubicin-induced apoptosis. The cells were transfected with pcDNA3.1, N17Rac1 + pcDNA3.1 or with N17Rac1 + WT-Rac1 plasmids. Viable cells were recovered after 24 h by ficoll gradient. The cells were then treated with doxorubicin for 24 h. Apoptosis was determined by annexin V staining and flow cytometry analysis. The results represent mean values ± SD from three independent experiments. *P < 0.05, **P < 0.01. **(E)** Rac1 inhibition blocks caspase-9 and -3 activation by doxorubicin. Jurkat cells were treated as indicated and after 12 h of doxorubicin treatment, the levels of native and active caspase-9 and -3 were determined by western blot analysis. β-actin was used as a loading control. The illustrated blots are representative of three independent experiments.

Growing evidence suggests that the GTPase Rac1 can have an important role in apoptosis^14–17^. We therefore assessed its implication in doxorubicin-induced apoptosis of myeloid U937 and lymphoblastic Jurkat cells. In the presence of the specific Rac1 inhibitor NSC23766, doxorubicin-induced apoptosis of U937 and Jurkat cells was reduced by 50% in both cell lines (Fig. 3A,B). NSC23766 showed no significant effect on leukemia cell survival when used alone. Transient expression of a dominant-negative form of Rac1 (N17Rac1) in U937 and Jurkat cells (Supplementary Fig. S2) also reduced doxorubicin-induced apoptosis by approximately 40% and 25% in comparison to cells transfected with a control plasmid (Fig. 3C,D). Exogenous expression of wild type Rac1 (Supplementary Fig. S2) abolishes the effect of N17Rac1 indicating that N17Rac1 specifically interferes with Rac1 signaling (Fig. 3C,D). The inhibition of Rac1 was associated with a significant reduction of doxorubicin-induced caspase-9 and-3 activation in both lymphoblastic (Fig. 3E) and myeloid (data not shown) leukemic cells. Thus, doxorubicin-induced apoptosis of these leukemic cells implicates Rac1.

### Collagen promotes doxorubicin resistance by inhibiting Rac1 activation

**Figure 4:**
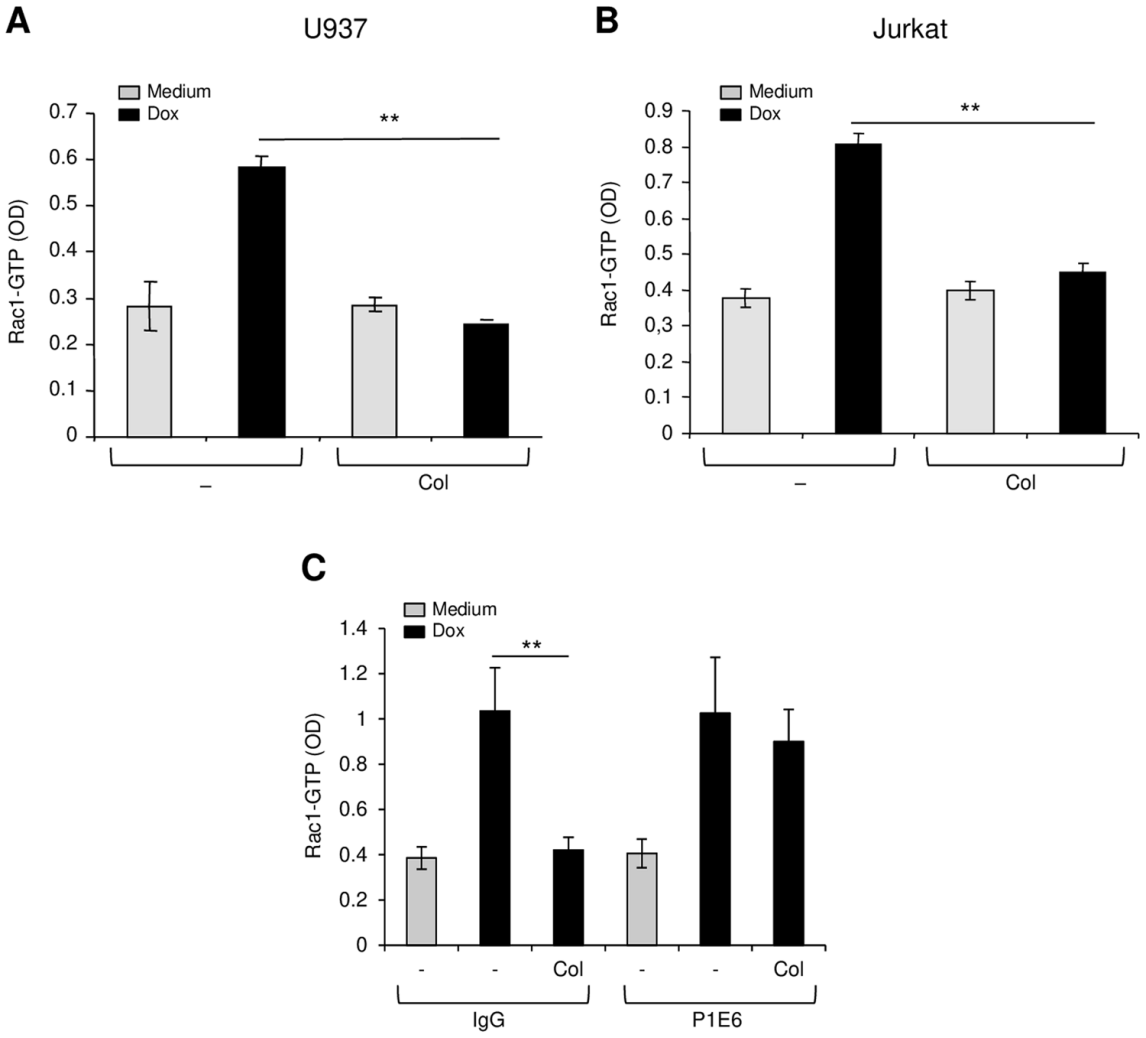
Collagen inhibits doxorubicin-induced Rac1 activation via α2β1 integrin. U937 (**A**) and Jurkat (**B**) cells were cultured on BSA (-) or on collagen (Col) and then treated or not with doxorubicin (Dox) for 3 h. The cells were harvested, lysed and Rac1 activation was determined by Rac1-GLISA assay. (**C**) The effect of collagen is mediated via α2β1 integrin. Jurkat cells were treated as above except that before their culture on collagen they were pretreated with 10 μg/ml of control IgG or with the blocking anti-α2 integrin mAb (P1E6). The results represent mean values ± SD from three independent experiments. **P < 0.01.

Our data indicated that Rac1 is an important pathway in doxorubicin-induced apoptosis of leukemia cells raising the question whether it is a targeted-event in collagen-mediated doxorubicin resistance in leukemia. Therefore, we assessed the effect of collagen signaling on doxorubicin-induced activation of Rac1 in leukemic cells. Exposure of myeloid U937 and lymphoblastic Jurkat leukemic cells to doxorubicin increases by two-fold the activation of Rac1 (Fig. 4A,B). Culturing these leukemic cells on collagen had no effect on Rac1 activation but abrogated doxorubicin-induced Rac1 activation. The effect of collagen on Rac1 activation was mediated via α2β1 integrin as the blocking anti-α2 integrin antibody reversed the effect of collagen (Fig. 4C). Thus, collagen/α2β1 integrin promotes doxorubicin resistance in leukemic cells via the inhibition of Rac1 activation.

### Collagen inhibits doxorubicin-induced Rac1 activation independently from drug efflux

**Figure 5:**
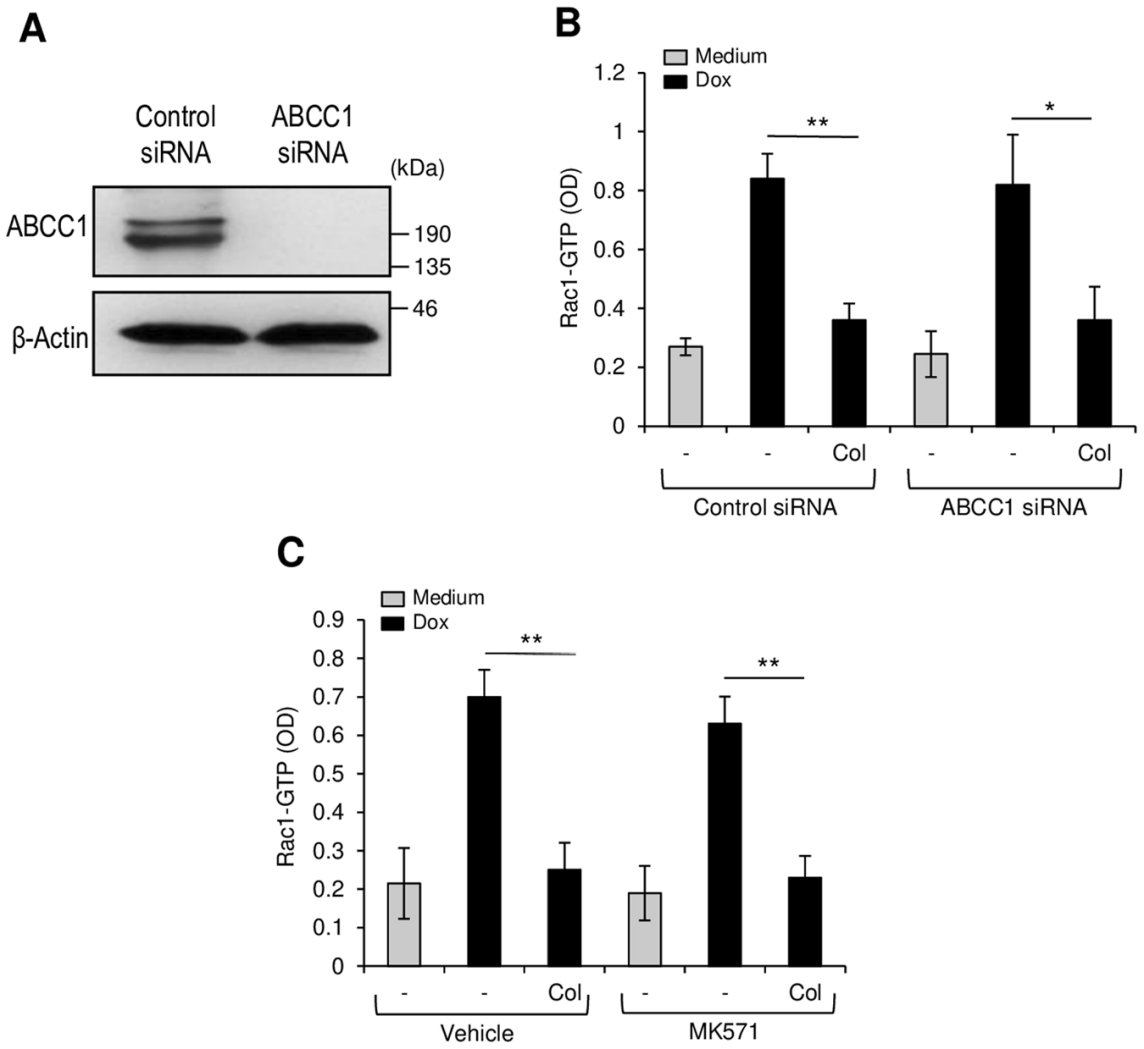
Collagen inhibits Rac1 activation independently from ABCC1. (**A**) ABCC1 protein levels in Jurkat T cells transfected with control and specific ABCC1 siRNA levels. The immunoblot is representative of three independent experiments. (**B**) ABCC1 silencing does not affect collagen-mediated Rac1 inhibition. Control and ABCC1 siRNA-transfected Jurkat T cells were cultured on BSA or on collagen and then treated with doxorubicin (Dox) for 3 h. The cells were harvested, lysed and Rac1 activation was determined by Rac1-GLISA assay. (**C**) The ABCC1 inhibitor MK571 does not affect collagen-mediated Rac1 inhibition in U937 cells. The cells were pretreated with the vehicle or MK571 (10 μM) for 1 h after which, the cells were cultured on BSA or collagen and then treated with Dox. Rac1 activation was determined by Rac1-GLISA assay. The results represent mean values ± SD from three independent experiments. *P < 0.05, **P < 0.01.

Since α2β1 integrin has been shown to enhance doxorubicin efflux by activating the drug transporter ABCC1^7^, it is possible that the observed inhibition of Rac1 could be the consequence of reduced drug concentration inside the cells. To examine this issue, we inhibited the transporter ABCC1 and then examined whether collagen still inhibits doxorubicin-induced Rac1 activation. The results indicate that ABCC1 knockdown in Jurkat cells with a specific siRNA (Fig. 5A) had no effect on the ability of collagen to inhibit doxorubicin-Rac1 activation (Fig. 5B). Furthermore, the ABCC1 specific inhibitor MK571 also did not affect collagen-induced Rac1 inhibition in U937 cells (Fig. 5C). Together these results indicate that collagen-induced drug efflux and inhibition of Rac1 are independent events.

### Collagen reduces Rac1-dependent DNA damage response

**Figure 6:**
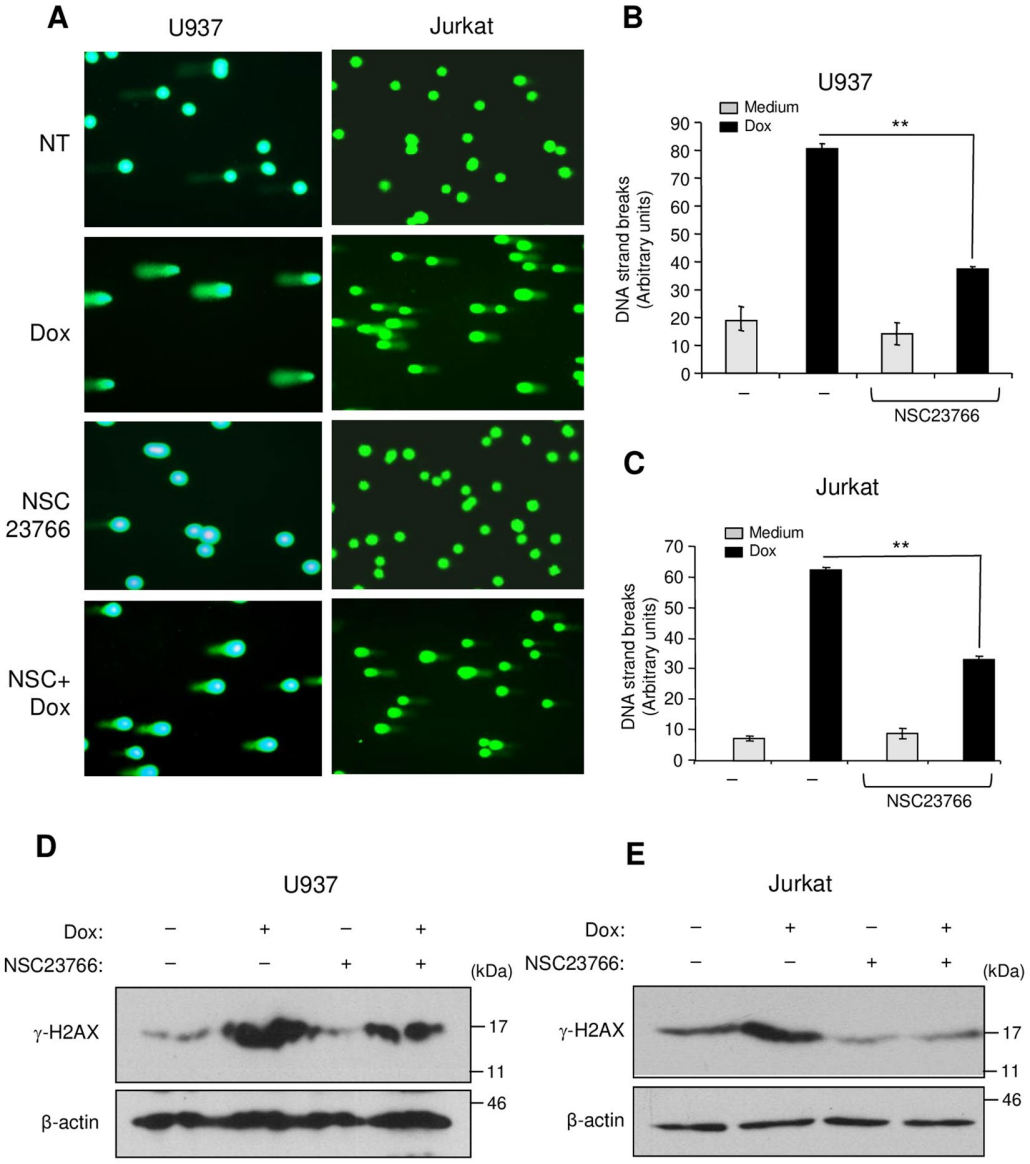
Rac1 inhibition reduces DNA damage intensity and H2AX phosphorylation induced by doxorubicin. (**A**–**C**) The cells were treated or not with doxorubicin (Dox) for 6 h in the presence or absence of the Rac1 inhibitor NSC23766 (NSC). Alkaline comet assay was performed and stained nucleoids were visualized by epifluorescence microscopy using FITC filter. (**A**) Representative fields corresponding to each treatment were photographed. (**B**,**C**) The intensity of DNA strand breaks in U937 and Jurkat cells was quantified using visual scoring as described under “Experimental procedures section”. The results represent mean values ± SD obtained from three independent experiments. **P < 0.01. (**D**,**E**) The cells were treated with doxorubicin in the presence or absence of NSC23766 as described above and the levels of phosphorylated H2AX (γ-H2AX) were determined by immunoblot analysis. The β-Actin blot was used as a loading control. Blots are representative of three independent experiments.

**Figure 7:**
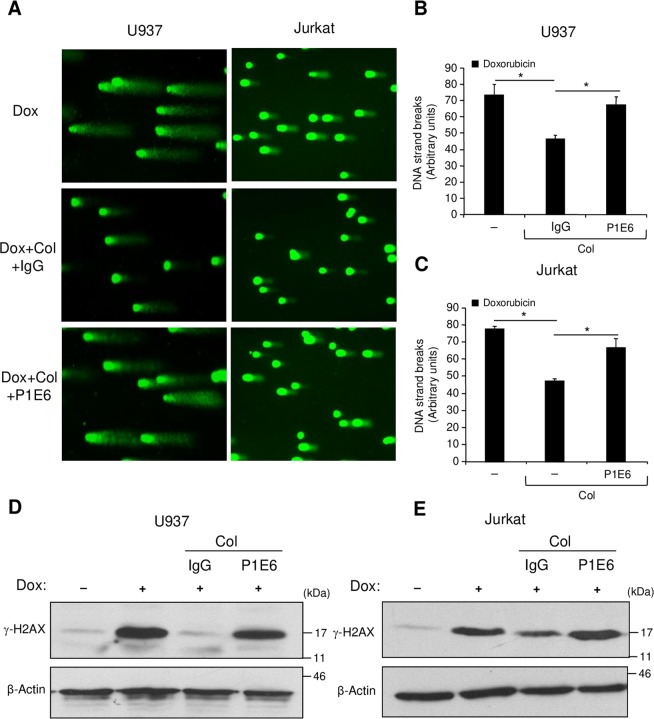
Collagen/α2β1 integrin inhibits doxorubicin-induced DNA damage and H2AX phosphorylation. (**A**–**C**) Collagen inhibits doxorubicin-induced DNA damage in U937 and Jurkat leukemic cell lines. The cells were pretreated for 1 h with 10 μg/ml of control IgG or with the blocking anti-α2 integrin antibody (P1E6) and then cultured on BSA or on collagen (Col) for 2 h. After removing cells in suspension, adherent cells were treated with doxorubicin (Dox) for 6 h. At the end, cells were harvested and the alkaline comet assay was performed and stained nucleoids were visualized by epifluorescence microscopy using FITC filter. **(A)** Representative fields corresponding to each treatment were photographed. (**B**,**C**) The intensity of DNA strand breaks was quantified using visual scoring as described under “Experimental procedures section”. The results represent mean values ± SD obtained from three independent experiments. *P < 0.05. (**D**,**E**) The cells were treated as indicated, and the levels of phosphorylated H2AX (γ-H2AX) were determined by immunoblot analysis. The β-Actin blot was used as a loading control. Blots are representative of three independent experiments.

Rac1 is important in the nucleus during genotoxic stress as it enhances the binding of topoisomerase poisons to their targets to form DNA double strand breaks and to activate the DNA damage response and subsequent apoptosis^19–21^. Therefore, we verified the implication of Rac1 in doxorubicin-induced DNA damage of leukemic cells. We used the comet assay, which allows evaluation of the intensity of the DNA strand breaks and we determined the phosphorylation levels of histone H2AX (γ-H2AX), which is an important component of the DNA damage response^30,31^. The presence of the Rac1 inhibitor NSC23766 reduced the intensity of DNA strand breaks (Fig. 6A–C) and diminished the phosphorylated levels of H2AX (Fig. 6D,E) indicating the implication of Rac1 in doxorubicin-induced DNA damage in leukemic cells. Importantly, adhesion of leukemic cells to collagen inhibited DNA damage induced by doxorubicin and this effect was blocked by the α2 integrin blocking antibody (Fig. 7A–C). Altogether, these data reveal that collagen can protect leukemic cells against doxorubicin-induced DNA damage at least by inhibiting Rac1.

### Rac1 is involved in doxorubicin-induced JNK activation and Mcl-1 downregulation

**Figure 8:**
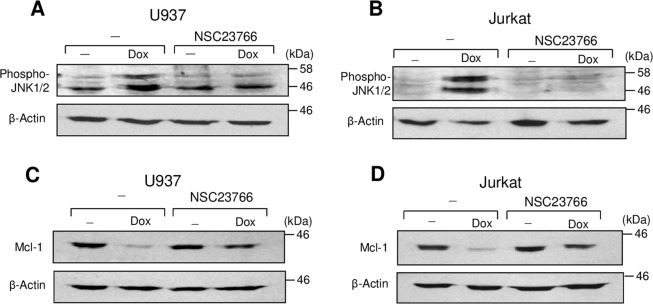
Rac1 is involved in doxorubicin-induced JNK activation and Mcl-1 downregulation. U937 and Jurkat cells were treated or not with doxorubicin (Dox) in the presence or absence of the Rac1 inhibitor NSC23766. After 8 h of treatment, the cells were lysed and the levels of phospho-JNK1/2 (A&B) and Mcl-1 (C&D) were determined by immunoblot analysis. The blots were stripped and reprobed with anti-β-actin antibody for equal loading. The blots are representative of three independent experiments.

One target of Rac1 during DNA-damage and induction of apoptosis is JNK^14–18^. The Rac1/JNK pathway has been involved in doxorubicin-induced apoptosis of cardiomyocytes and human cancer cells^18,32,33^. Previously, we found in T-ALL cells that doxorubicin-induced JNK activity promoted apoptosis by downregulating the levels of anti-apoptotic protein Mcl-1, and collagen inhibited JNK activation and restored Mcl-1 levels^6^. Herein, we demonstrate that Rac1 is involved in doxorubicin-induced JNK activation and Mcl-1 downregulation in both myeloid and lymphoblastic leukemic cells (Fig. 8) indicating that collagen/α2β1 integrin inhibits doxorubicin-induced JNK activation and Mcl-1 downregulation likely by preventing Rac1 activation.

## Discussion

The mechanisms by which integrin/ECM interactions regulate leukemia chemoresistance are not fully understood. Previous studies have shown the importance of fibronectin-binding integrins α4β1 and α5β1 in the chemoresistance of AML cells^25^. However, clinical investigations found that α4β1 integrin levels were either not associated with relapse and overall survival or predicted a better outcome in AML patients^34,35^. Herein, we found no association of α4 integrin levels with overall survival and we determined that higher levels of α5 integrin are associated with a better prognosis in an independent AML cohort. Together these results suggest that fibronectin-binding integrins may not be critical or sufficient to confer drug resistance and predict clinical outcome in AML. Along these lines, we show that collagen/α2β1 integrin, similar to its role in T-ALL cells^6,7^, promotes doxorubicin resistance of AML cell lines and clinical analysis of an AML cohort indicates that high α2 integrin levels correlate with poor prognosis. A recent study reported similar findings with regard to α2 integrin in independent AML cohorts^36^. The association of α2 integrin with poor prognosis and relapse in AML patients could be explained by the results reported herein showing that α2 integrin promotes doxorubicin resistance in AML. This is further supported by previous findings showing that HL-60 cell adherence to collagen also promotes their protection against apoptosis induced by Ara-C and irradiation^37^.

In previous studies, fibronectin did not protect T-ALL cell lines and blasts from chemotherapy-induced apoptosis^6,7^, but crosslinking of α4β1 and α5β1 integrins has the capacity to promote Jurkat T cell chemoresistance^38^. In contrast, the authors of this study used recombinant fibronectin ligands but not the full fibronectin molecule. However, microarray analysis in large cohorts of pediatric T-ALL patients demonstrated that genes encoding adhesion molecules are the best predictors of relapse^39^. Interestingly, α2β1 integrin but not fibronectin-binding integrins is among the adhesion molecules identified. In addition, we recently reported that the blockade of β1 integrin overcomes doxorubicin resistance in a model of T-ALL xenograft^40^. While additional studies are needed to sort out the role of each α integrin subunit in leukemia chemoresistance, our findings argue for an important role for the collagen-binding integrin α2β1 in the chemoresistance of both AML and T-ALL. However, β3 integrins may also be important for leukemogenesis and chemoresistance in AML^41^.

Our results showed that doxorubicin-induced apoptosis of both myeloid and lymphoblastic leukemic cells requires Rac1 activity, which is inhibited by collagen/α2β1 integrin signaling, thus inducing doxorubicin resistance. Rac1 belongs to the family of small GTPases and is involved in cytoskeleton remodeling. Integrins can activate Rac1 during cell adhesion and migration^12,13^, but collagen alone had no effect on Rac1 activation in the leukemic cells examined herein. Similarly, the collagen-binding integrin α1β1 reduces the generation of reactive oxygen species in mesangial cells by negatively regulating epidermal growth factor receptor-mediated Rac1 activation^42^. Together, these findings indicate that in some settings, integrins block activation of Rac1.

Rac1 is involved in drug-induced DNA damage response^19,20^ leading to either DNA repair or to the induction of cell death^22^. We showed that Rac1 is necessary for the induction of DNA damage by doxorubicin as demonstrated by the comet assay and the induction of phosphorylated H2AX, which is a hallmark of DNA damage response^30,31^. Collagen/α2β1 integrin decreased the intensity of DNA damage and inhibited the levels of γ-H2AX; therefore interfering with DNA damage signaling induced by doxorubicin. It has recently been shown that β1 integrin can promote drug and radioresistance of cancer cells by directly enhancing DNA repair^9–11^. Our results suggest that the effect of α2β1 integrin on the inhibition of DNA damage occurs indirectly via Rac1 inhibition. However, we cannot exclude the possibility that α2β1 integrin also affects directly the DNA repair pathway. Thus, we propose that α2β1 integrin protects leukemic cells from genotoxic agents and subsequent apoptotic death at least by inhibiting Rac1 activation.

Collagen-induced Rac1 inhibition in doxorubicin-treated cells also contributes to explain the previously reported antagonizing effects of collagen on doxorubicin-induced JNK activation and Mcl-1 downmodulation^6^ as these events depend on Rac1 (Fig. 8). Accordingly, by inhibiting Rac1, collagen/α2β1 integrin signaling inhibits drug-induced DNA damage and JNK activation, restores Mcl-1 levels thereby promoting leukemia chemoresistance.

Although α2β1 integrin enhances doxorubicin efflux in leukemic cells^7^, this does not seem to contribute to the observed inhibition of Rac1 since ABCC1 inhibition did not alter the ability of collagen to inhibit Rac1. These results indicate that enhancement of doxorubicin efflux via ABCC1 and inhibiting Rac1 activation are both critical and independent events in collagen-α2β1 integrin-mediated doxorubicin resistance in leukemic cells.

Rac1/JNK has been involved in doxorubicin-induced toxicity in cardiomyocytes^18^. In support of our study, was the finding that depletion of focal adhesion kinase (FAK) enhances doxorubicin cardiotoxicity^43^. Activation of FAK by collagen/α2β1 integrin signaling also occurs in Jurkat cells^44,45^. Cardiomyocytes express α1β1 but not α2β1 as a collagen-binding integrin^46^. Thus, the absence of α2β1 integrin could be among the causes explaining the increased sensitivity of cardiomyocytes to doxorubicin.

In conclusion, our study discloses α2β1 integrin as an important pathway of acute leukemia cell resistance to anthracycline-induced genotoxic stress through its capacity to inhibit Rac1-induced DNA damage and subsequent apoptosis. From a translational perspective, combining anthracycline drugs and α2β1 integrin blockers or the design of novel agents that can induce leukemia cell death independently from the Rac1 pathway might be helpful in preventing the emergence of drug-resistant leukemic cells.

## Materials and Methods

### Reagents and antibodies

Collagen type I, fibronectin and doxorubicin were purchased from Millipore-Sigma (Billerica, MA, USA). The ABCC1 inhibitor, MK571, was from Calbiochem (San Diego, CA, USA). The Rac1 inhibitor (NSC23766) was obtained from Tocris Bioscience (Ellisville, MO, USA). Recombinant human VCAM-1/CD106 protein was from R&D systems (Minneapolis, MN, USA). The anti-caspase-3 (E-8) that detects the native and the active fragments of caspase-3, anti-Mcl-1 (22) and anti-β-actin (C-2) antibodies were from Santa Cruz Biotechnology (Santa Cruz, CA, USA). The anti-caspase-9, which detects the native and active fragments of caspase-9 and anti-Phospho-JNK (Thr183/Tyr185) (G9) antibodies were from Cell Signaling Technologies (Beverly, MA, USA). The anti-phosphorylated-histone H2AX (Ser 139) (JBW301) and anti-ABCC1 (QCRL-1) antibodies were purchased from Millipore-Sigma (Billerica, MA, USA). PE-conjugated anti-human α2 integrin (clone 12F1), PE-conjugated anti-human α1 integrin (clone SR84) and APC-conjugated anti-β1 integrin (clone MAR-4) and isotypic control antibodies were from BD Biosciences (San Diego, CA, USA). The anti-β1 integrin (clone 4B4) and anti-α2 integrin (PEI6) blocking antibodies were purchased from Beckman Coulter (Brea, CA, USA) and Millipore-Sigma (Billerica, MA, USA) respectively. The antibody against the c-Myc tag (Millipore-Sigma) was obtained from Sylvain Bourgoin (Laval University).

### Leukemia cell lines and cell culture

The acute myeloid leukemia (AML) cell lines U937, HL-60, PLB-985 and the T-ALL cell line Jurkat were from ATCC (Manhasset, VA, USA). Cells were maintained in RPMI 1640 medium supplemented with 10% of fetal bovine serum (FBS), 2 mmol/l of glutamine and 100 units/mL of penicillin and streptomycin.

### Cell surface expression of integrin subunits

The cells were first incubated on ice for 1 h with inactivated human serum to block putative Fcγ receptors. The cells were then washed with PBS and stained with 10 μg/ml of PE-conjugated antibodies against human α1 and α2 integrins, and APC-conjugated antibody against human β1 integrin or with their corresponding isotypic antibodies for 30 minutes on ice. The cells were washed with PBS and analyzed by flow cytometry (FACSCalibur, BD Biosciences).

### AML clinical cohort

The Leucegene cohort of 430 patient samples with clinical data has been RNA-sequenced in previous projects (www.leucegene.ca) and already published^26^.

### Matrix coating and determination of Apoptosis

48-well plates (Falcon^®^, Fisher Scientific Inc, USA) were coated overnight with 1 mg/ml of collagen type I, fibronectin or with bovine serum albumin (BSA) (1%) at room temperature under the drying air of the hood as previously described^47,48^. Leukemic cells (5 × 10^5^) in 500 μl of serum-free medium containing 50 μg/ml BSA were seeded into coated wells. After 2 h incubation at 37 °C, the wells were washed gently to remove non-adherent cells. The remaining cells were then treated with doxorubicin in RPMI medium containing 2.5% serum. Apoptosis was determined after 24 h by annexin V staining and flow cytometry analysis using the FACSCalibur cytometer (BD Biosciences).

### Caspase activation, Mcl-1 levels, and JNK and H2AX phosphorylation

Caspase activation, H2AX and JNK phosphorylation, and Mcl-1 protein levels were determined by immunoblot analysis using specific antibodies as we previously described^6^. β-actin was used as a loading control.

### Plasmids and transient cell transfection

The plasmids encoding c-Myc-tagged forms of wild type Rac1 (WT-Rac1) and dominant-negative Rac1 (N17-Rac1) were previously described^49^ and were obtained from Josée N. Lavoie (Laval University). Two million cells were transfected with a total of 6 μg of the different plasmids using Amaxa nucleofector apparatus (Program C016) according to the manufacturer’s instructions. After 48 h of transfection, viable cells were recovered by ficoll gradient and used in subsequent experiments. Transfection efficiency was verified by western blot analysis using anti-c-Myc tag antibody.

### Measurement of Rac1 activity

Commercial Rac-1 G-LISA™ kit customized to capture and quantify Rac1-GTP was purchased from Cytoskeleton, Inc (Denver, CO, USA). Rac1-GTP activity was assessed on treated and untreated cell lysates according to the manufacturer’s instructions.

### ABCC1 siRNA

Jurkat T cells were transfected with control and validated ABCC1 siRNA; a mix of four siRNA sequences (L-007308-00-0005; Dharmacon, Lafayette, CO) by the nucleofector method as we previously described^7^.

### Comet assay (single-cell gel electrophoresis) and quantification of DNA strand breaks

The intensity of DNA damage was evaluated using the Oxiselect^TM^ Comet assay kit purchased from Cell Biolabs, Inc (San Diego, CA, USA). The alkaline version of the test was used for its more sensitivity than the neutral version and was performed as described by the manufacturer. Ethidium bromide-stained nucleoids were visualized by epifluorescence microscope (Olympus BX51) using FITC filter (excitation: 460–500 nm, emission 510–560 nm). Cellular nuclei were photographed using the CoolSnap HQ digital camera. The intensity of DNA damage was determined as described previously by Park *et al*.^50^. 100 comets/slide were scored visually and classified according to the tail intensity and assigned a value of 0, 1, 2, 3, or 4 (0 indicates undamaged, 1 indicates slightly damaged, 2 indicates moderately damaged, 3 indicates severely damaged, and 4 indicates very severely damaged). The total score for 100 comets was again divided by factor of 5 to yield an arbitrary value ranging from 0 (if the counted a hundred comets are all undamaged) to 80 (if the counted 100 comets are all very severely damaged).

### Statistical analysis

Statistical analysis was performed using two-tailed Student’s t-test. Significance of Kaplan-Meier survival curves was determined by the using log-rank test. *P*-values < 0.05 were considered significant.

## Supplementary information

Supplementaries Fig. S1 and S2
