# Supplementary material for: Cell adhesion to collagen promotes leukemia resistance to doxorubicin by reducing DNA damage through the inhibition of Rac1 activation
Source: Sci Rep. 2019 Dec 19;9:19455. doi: 10.1038/s41598-019-55934-w (PMC6923425; doi:10.1038/s41598-019-55934-w)
Supplement: Supplementary file 1 — Supplementaries Fig. S1 and S2 [file 41598_2019_55934_MOESM1_ESM.pdf]

Supplementary file

Cell adhesion to collagen promotes leukemia resistance to doxorubicin by reducing DNA damage through the inhibition of Rac1 activation.

Dalila Naci [#], Sofiane Berrazouane, Frédéric Barabé[1] and Fawzi Aoudjit[2*]

Centre de recherche du CHU de Québec-Université Laval, Axe des maladies infectieuses et immunitaires.

[1] Département de Médicine, Faculté de Médecine, Université Laval, Québec, Canada

[2]Département de Microbiologie-infectiologie et Immunologie, Faculté de Médecine, Université Laval, Québec, Canada.

# Present Address: The Hospital for Sick Children, University of Toronto, Toronto, Canada.

Additional File 1

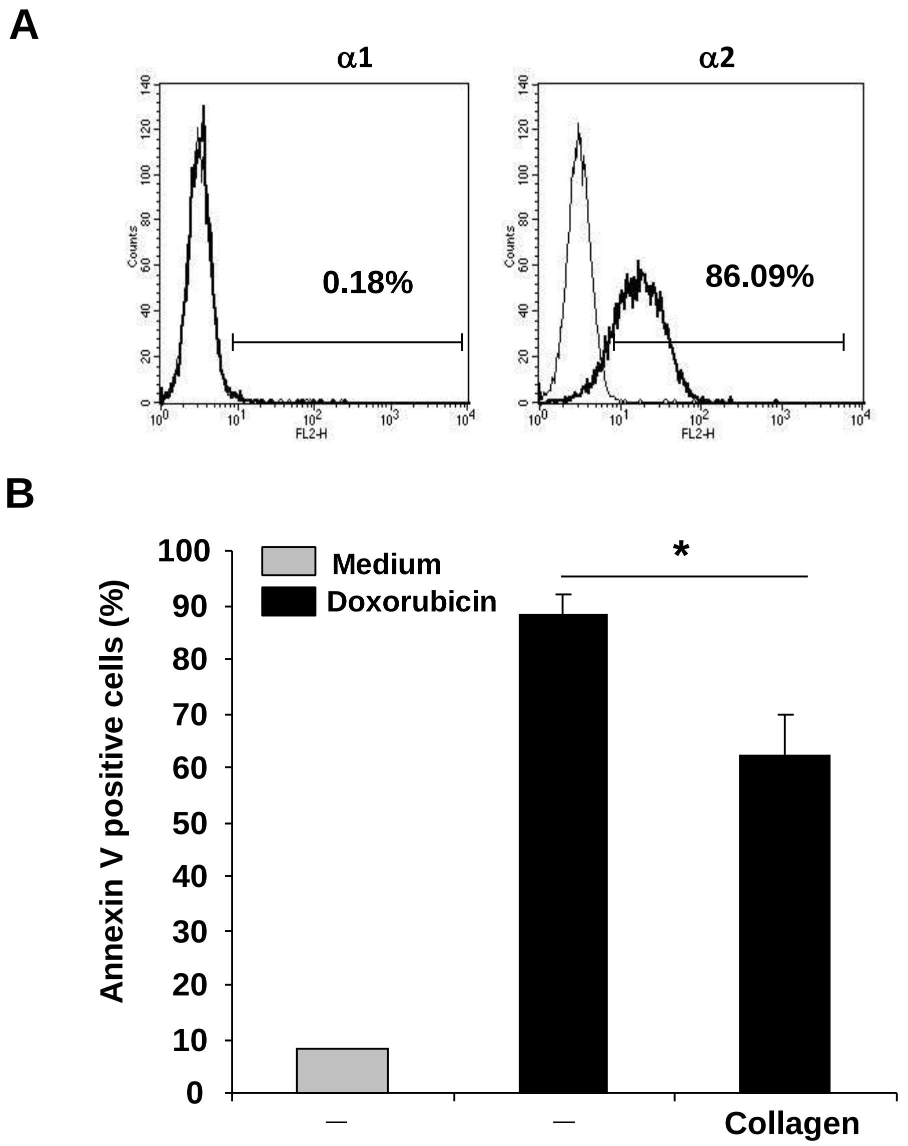

Figure S1: Collagen promotes PLB-985 AML cell protection against doxorubicin-induced apoptosis. A) PLB-985 cells express α2β1 but not α1β1, collagen- binding integrin. Expression levels of α1 and α2 integrin subunits were determined by flow cytometry using PE-conjugated specific antibodies. **B)** Collagen reduces doxorubicin-induced apoptosis of PLB-985 cells**.** Cells were cultured on collagen for 4h and then treated with doxorubicin for 24h. Apoptosis was evaluated by AnnexinV-FITC staining and FACS analysis. The results represent the mean values± SD from two independent experiments. *$p<0.05$.

Additional File 2

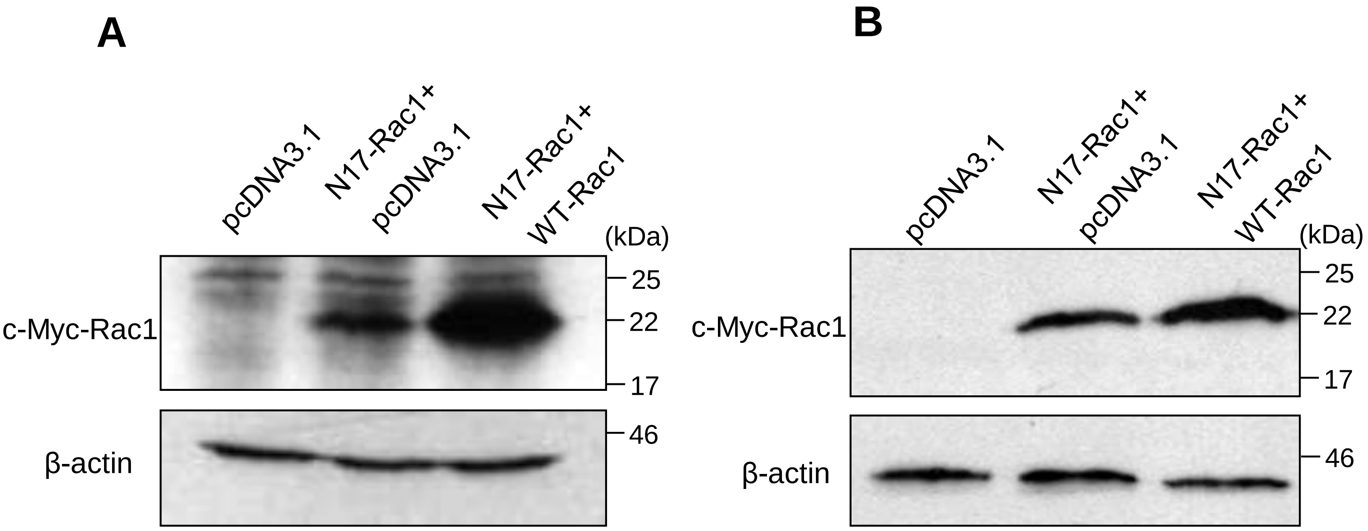

Figure S2: U937 (A) and Jurkat cells (B) were co-transfected with c-Myc-tagged dominant negative Rac1 (N17Rac1) and wild-type Rac1 (WT-Rac1) plasmids using nucleofector. Transfections with pcDNA3.1 plasmids were used as controls. Viable cells were recovered 24h after transfection by ficoll gradient. c-Myc expression was determined by western blot analysis using the anti-c-Myc specific antibody. β-actin was assessed to control equal loading.
